# The Neutrophil to Lymphocyte Ratio Predicts the Response to Neoadjuvant Chemotherapy in Luminal B Breast Cancer

**DOI:** 10.31557/APJCP.2019.20.7.2209

**Published:** 2019

**Authors:** Mauricio Rivas, Francisco Acevedo, Francisco Dominguez, Hector Galindo, Mauricio Camus, David Oddo, Alejandra Villarroel, Dravna Razmilic, Jose Peña, Matias Muñoz-Medel, Maria Elena Navarro, Alejandra Perez-Sepulveda, Lidia Medina, Tomas Merino, Juan Briones, Alexis Kalergis, Cesar Sanchez

**Affiliations:** 1 *Department of Hematology and Oncology, *; 2 *Department of Oncological and Maxillofacial Surgery,*; 3 *Department of Anatomy and Pathology, *; 4 *Department of Radiology, School of Medicine, *; 6 *Millennium Institute on Immunology and Immunotherapy, Department of Molecular Genetics and Microbiology, Faculty of Biologic Sciences, Pontificia Universidad Catolica de Chile,*; 5 *Centro de Cancer, Red de Salud UC-CHRISTUS, Santiago, Chile. *

**Keywords:** Neutrophil to lymphocyte ratio- breast cancer- luminal subtype- neoadjuvant chemotherapy

## Abstract

**Objective:**

Tumor response to neoadjuvant chemotherapy (NAC) in breast cancer (BC) patients is a predictor for overall survival. The aim of our study was to determine a relationship between the neutrophil to lymphocyte ratio (NLR) prior to NAC, BC subtypes and the probability of a pathologic complete response (pCR).

**Materials and Methods:**

Medical records were collected retrospectively from Centro de Cancer at Red Salud UC-Christus. Clinical data collected included peripheral blood cell counts, BC subtype at diagnosis and the pathology report of surgery after chemotherapy.

**Results:**

A total of 88 patients were analyzed. Approximately, a 25% had a pCR, and displayed a significant correlation between BC subtype and pCR (p= 0.0138 Chi2); this was more frequent in epidermal growth factor receptor type 2 (HER2) enriched subtype patients (54%). Luminal B BC patients with a pCR had significantly lower NLR levels (t test, p= 0.0181).

**Conclusions:**

HER2-enriched tumors had a higher probability of pCR. In Luminal B tumors, NLR had a statistically significant relationship with the probability of pCR. In this subtype, NLR could be a useful biomarker to predict tumor response to NAC. Further studies including other clinical parameters for systemic inflammation such as platelet counts, intratumoral NLR or body mass index could help identify patients that would get the most benefit from NAC.

## Introduction

Breast cancer (BC) is the first cause of cancer death among Chilean women. Data from 2018 indicate a crude incidence rate of 58.7 and a crude mortality rate of 18.4 per 100,000 women, respectively (Bray et al., 2018). As occurs in most malignancies, inflammation and immune status are key determinants of carcinogenesis and cancer progression. Indeed, lymphocyte tumor infiltration and inflammatory processes have been associated to BC subtypes and prognosis (Savas et al., 2016). A number of inflammation and immune infiltration markers have been previously described. However, their use in the clinic is limited mainly due to high costs and a lack of validation for this setting. A previous report by our group (Mimica et al., 2016) demonstrated that the Neutrophil to Lymphocyte Ratio (NLR), a measure of systemic inflammation routinely used in the clinic, was associated to aggressive BC subtypes, including epidermal growth factor receptor type 2 (HER2) overexpressing or triple negative tumors; predicting survival in the first. An accurate assessment of residual tumor disease after NAC allows the identification of those patients with a pathological complete response (pCR). These patients are more likely to display long-term better survival levels; suggesting pCR is a surrogate marker for overall survival (OS), especially on aggressive BC subtypes. Here, we assessed NLR levels and pathologic response rates in patients receiving NAC. 

## Materials and Methods

*Patients*

This was a retrospective study. Medical records from BC patients receiving NAC over the period 1997-2017 were obtained at the Centro del Cancer in the Pontificia Universidad Catolica de Chile and the Red de Salud UC CHRISTUS in the city of Santiago, Chile. Inclusion criteria were: 1) to receive at least one cycle of cytotoxic therapy; and 2) available medical records including evidence from pathology record in order to determine tumor response rate to therapy. Exclusion criteria were patients without cytotoxic therapy treatment, no pathology records and/or follow-up information. 

*Neutrophil to Lymphocyte Ratio (NLR)*

The NLR for each patient was calculated from complete blood counts (hemograms) obtained at a date close to the date of diagnosis and within three months prior to the start of NAC.

**Figure 1:**
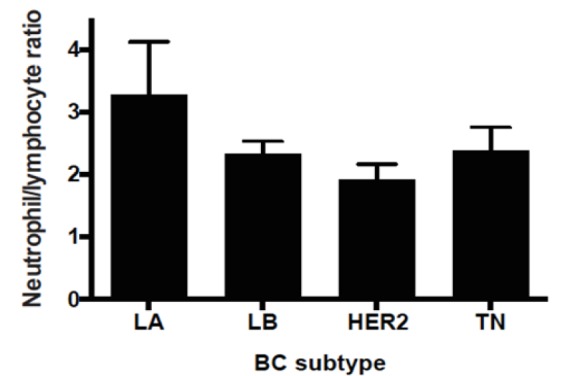
Neutrophil to Lymphocyte Ratio by Breast Cancer Subtype

**Figure 2:**
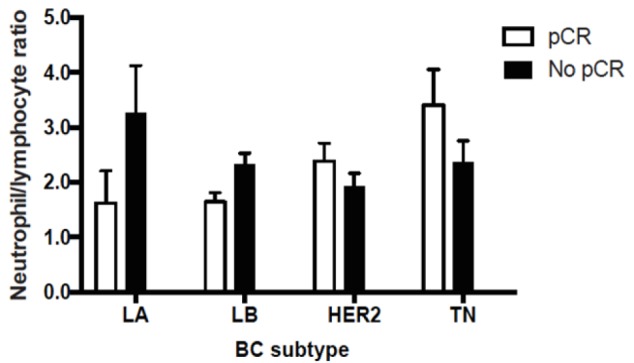
Neutrophil to Lymphocyte Ratio Levels with or without pCR by Breast Cancer Subtype

**Figure 3:**
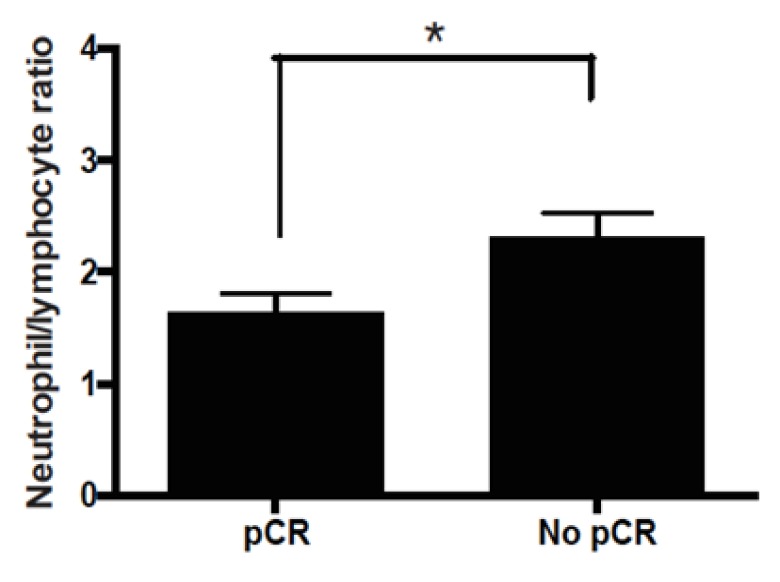
Neutrophil to Lymphocyte Ratio Levels in Luminal B Subtype. * p<0.005

**Table 1:** Basic Characteristics of Chilean Breast Cancer Patients from Study Population

Characteristics	Patients (n=88)	%
Age (median-range)	49 (27-75)	
Stage		
I	5	5.7
II	32	36.4
III	49	55.7
IV	2	2.3
Histology		
Ductal	84	95.5
Lobular	4	4.5

**Table 2:** Pathological Complete Response Status Frequency by BC Subtype

Subtypes	pCR(%)	No pCR%	Total
Luminal A	2 (9)	19	21
Luminal B	8 (23)	26	34
HER2 enriched	6 (54)	5	11
Triple negative	6 (28)	16	21
Total	22 (25)	66	88

*BC subtypes*

Histological type, tumor size, histological grade (HG) and lymph node status were determined for all patients included in the study. Patients with a HG 1 or 2 were grouped and classified as low proliferation rate tumors. Estrogen receptor (ER), progesterone receptor (PR) and epidermal growth factor receptor type 2 (HER2) status were determined by immunohistochemistry (IHC). A ≥ 1% of positive tumor cell nuclear stain was established as cutoff value for ER and PR positivity. For HER2, a stain of +++ by IHC was considered positive. For most HER2 cases categorized as ++ or undefined an additional test by in situ hybridization was performed. Clinical status at diagnosis was determined according to the guidelines of the American Joint Committee on Cancer Staging 7^th ^edition (Edge, 2009). Thereby, tumors were classified into 4 subtypes: Luminal A (LA), Luminal B (LB), HER2-enriched or Triple Negative (TN) as described previously.

*Tumor response*

A pCR was defined as the absence of invasive tumor in the breast tissue (independent from the presence of an in situ component) or in axillary lymph nodes (ypT0/is-ypN0). Patients with a partial or no responses were classified as “no-pCR”. 

*Statistical analysis*

The Chi-square (χ^2^) or the Fisher’s exact tests were used to evaluate categorical variables. The pCR/NLR correlations were assessed by Welch corrected Student’s t-test, BC subtype/pCR correlations used χ^2^. ANOVA was used for comparisons among groups. Statistical significance was set at p≤0.05. All data were analyzed in IBM^®^ SPSS^®^ v.21. 

## Results

A total of 88 patients were included into this study. These patients had hemogram reports within a 3-month window prior to NAC in the period 2004-2018. Patient clinical characteristics are summarized in Table 1. Median age was 49 years (range: 27-75). Distribution of patient tumor stage prior to treatment was: Stage I 5.7%, stage II 36.4%, stage III 55.7% and stage IV 2.3%. A 95.5% of tumors were classified as ductal; the remaining were lobular (4.5%). Median NLR was 2 (range: 0.56-15.8) and higher NLR was associated to aggressive BC subtypes (HER2-enriched or TN). NLR by subtype was: LA: 1.75± 3.4; LB: 1.81± 0.93; HER2+: 2.14± 0.73; TN: 2.25± 1.55. These differences were not statistically significant (Figure 1, p=0.3622 by ANOVA). 

A 25% of NAC-treated patients displayed pCR. We found a significant correlation between BC subtype and tumor response. The best correlation was observed in HER2+ tumors (p=0.0138, Table 2).

Luminal subtype patient tumors (LA or LB) that displayed pCR had lower NLR levels against those without pCR. Conversely; aggressive subtypes (HER2-enriched or TN) with a pCR had higher NLR levels that did not reach statistical significance (Figure 2, p=0.32 by Student’s t-test). Finally, an analysis by BC subtype demonstrated a significant correlation between NLR and pCR in LB patients; in this subtype lower NLR levels were associated to a higher probability of pCR (p=0.00181; Figure 3).

## Discussion

The goal of our study was to identify a relationship between NLR levels prior to NAC and tumor response rates in BC patients. Although we did not find a relationship between these parameters in all analyzed patients, we found that lower NLR levels prior to NAC were associated to higher probability of pCR in LB patients. Interestingly, a similar trend that associates lower NLR to higher pCR is observed among luminal subtypes (LA or LB). Conversely, in non-luminal subtypes (HER2-enriched or TN) lower NLR levels are more associated to lower pCR probability. As occurs with our abovementioned results these differences did not reach statistical significance probably due to our small sample size. However, these trends open the possibility for future studies on NLR, pCR and BC subtypes, emphasizing the heterogeneity of BC. 

Previous studies indicate that the absence of post NAC residual tumor disease (pCR) correlates with survival rates, especially in aggressive BC subtypes (Savas et al., 2016). Thus, an assessment of tumor response opens the possibility for an objective measure of clinical interventions and the role of predictive and prognostic markers.

Previous reports by our group (Acevedo et al., 2015) and by others (Rouzier et al., 2005) suggest the use of BC subtypes as a pCR predictor factor; also a post NAC pCR is associated to OS (Spring et al., 2017).

Several studies have established NLR as an independent predictor for OS, cause-specific mortality and disease-free survival (DFS) (Ou et al., 2017; Ozyalvacli et al., 2014), and as a marker for systemic inflammation. Cancer-associated inflammation derives from the tumor microenvironment and can be assessed by biomarkers or used as a prognostic factor across many malignancies, including BC. Besides NLR, other inflammation related biomarkers have been evaluated in BC progression including platelet counts, body mass index or ultra-sensitive C-reactive protein (Sylman et al., 2018). As a marker for systemic inflammation, elevated NLR levels are also present in infection-derived inflammatory processes. A study in patients with Helicobacter pylori infection found a significant correlation with the severity of gastritis and NLR levels (Farah and Khamisy-Farah, 2014), this represents a potential recall bias in our study. However, since our patients were set to undergo chemotherapy, it is unlikely that they were infected as one of the main conditions to receive cytotoxic treatment is to effectively eradicate previous symptomatic infections. 

Previously, we demonstrated that NLR levels within 3 months prior to the start of therapy were associated to survival in a cohort of 130 stage I-III BC patients that included 81 TN and 49 HER2+. Lower survival rates on HER2+ BC were associated to elevated NLR levels (median NLR was 3.21 for deceased and 1.79 for alive patients). Here, we described a similar association between higher NLR and mortality rates for all patients (death HR=2.56, p=0.01)3. A study by Ozyalvacli (2014) demonstrates higher NLR levels in malignant breast tumors, compared to non-malignant tumors and postulates a cutoff NLR value of 2.96. Similarly, Azab (2012) suggested that NLR > 3.3 is associated to early and late mortality in a cohort of 316 treated BC patients. A large meta-analysis that involved 5 studies and a total of 3350 patients reported a significant association between NLR and OS (HR de 2.28 95% CI= 1.08-4.80. p<0.001) (Chen et al., 2015). Moreover, another meta-analysis by Wei (2016) that included 12 studies and 7951 patients demonstrated similar results. More recently, a study by Ethier (2017) involving 8563 patients reports HR=2.5 for overall mortality with a NLR > 3 and HR=1.7 for the inverse of DFS, independent from disease stage (early or metastatic). 

A study in patients with post-surgery recurrent disease found a 0.59 increase in NLR (relative to its value at diagnosis) associated to recurrence (Iwase et al., 2017). On the other hand, a study that assessed NLR and the Lymphocyte to Monocyte Ratio (LMR) in 1570 patients concluded that NLR > 2 and LMR > 4.8 were associated to better DFS. Also, a low NLR predicted for better OS and DFS (HR was 1.6 and 1.5, respectively) (Jia et al., 2015). Other studies also demonstrated a NLR/DFS association (Orditura et al., 2016).

A number of studies have assessed NLR as a predictor of tumor response by NAC. Chen (2016) demonstrated that pCR was higher among women with a NLR > 2.06 prior to chemotherapy (24 vs 14%; p <0.05) in a Chinese cohort (n=215). In contrast, Erylmaz (2014) did not find an association between NLR and pCR in a Turkish cohort (n=78). Similarly, Suppan (2015) evaluated 247 Austrian women that received chemo or hormone neoadjuvant therapy and did not find a significant association. Asano (2016) reported that a NLR < 3 was associated to better treatment response in a Japanese cohort (n=177). Finally, Qian et al., (2018) found an association between NLR < 2.15 and a higher pCR in a Chinese cohort (n=180). 

The study of other systemic inflammation markers such as intratumoral NLR, platelet, monocyte or lymphocyte counts along with a standardization of these measures and the use of a larger sample size will improve and further validate the relationship between NLR (or other inflammation markers) and pathological response. 

In conclusion, our results suggest a prognostic role of NLR; elevated NLR levels could identify patients at a higher risk or with a poor response to NAC and consequently worse prognosis. Hence, NLR is a low cost, accessible and easy to interpret assay, and these characteristics make it a valuable marker in the clinic to assess prognosis in LB patients. Despite this, the association of NLR to other inflammation markers could further validate it as an effective predictor. 
